# Rapid metabolism increases the level of 2,4-D resistance at high temperature in common waterhemp (*Amaranthus tuberculatus)*

**DOI:** 10.1038/s41598-019-53164-8

**Published:** 2019-11-13

**Authors:** Chandrima Shyam, Amit J. Jhala, Greg Kruger, Mithila Jugulam

**Affiliations:** 1 Department of Agronomy, Kansas State University, 2004 Throckmorton Plant Sciences Center, 1712 Claflin Road, Manhattan, KS 66506 USA; 2 Department of Agronomy and Horticulture, University of Nebraska-Lincoln, 202 Keim Hall, Lincoln, NE 68583 USA; 3 University of Nebraska-Lincoln, North Platte, NE 69101 USA

**Keywords:** Abiotic, Heat

## Abstract

Common waterhemp emerges throughout the crop growing season in the Midwestern United States, and as a result, the seedlings are exposed to a wide range of temperature regimes. Typically, 2,4-D is used in the Midwest to control winter annual broad-leaf weeds before planting soybean and in an early post-emergence application in corn and sorghum; however, the evolution of 2,4-D-resistant common waterhemp in several Midwestern states may limit the use of 2.4-D for controlling this problem weed. Moreover, temperature is one of the crucial factors affecting weed control efficacy of 2,4-D. This research investigated the effect of temperature on efficacy of 2,4-D to control 2,4-D susceptible (WHS) and -resistant (WHR) common waterhemp. Do se-response of WHS and WHR to 2,4-D was assessed at two temperature regimes, high (HT; 34/20 °C, d/n) and low (LT; 24/10 °C, d/n). Whole plant dose response study indicated an increased level of 2,4-D resistance in WHR at HT compared to LT. Additional investigation of the physiological mechanism of this response indicated that both WHS and WHR common waterhemp plants rapidly metabolized ^14^C 2,4-D at HT compared to LT. In conclusion, a rapid metabolism of 2,4-D conferred increased level of resistance to 2,4-D in WHR at HT. Therefore, application of 2,4-D when temperatures are cooler can improve control of 2,4-D resistant common waterhemp.

## Introduction

Common waterhemp [*Amaranthus tuberculatus* (Moq.) Sauer] is one of the most troublesome weeds that can cause extensive yield loss in major agronomic crops in the Midwestern United States. Season-long interference of common waterhemp can result in up to 56% and 74% yield loss in soybean^1^ and corn^2^, respectively. Biological characteristics of common waterhemp, such as continuous emergence pattern, high fecundity, and adaptability to diverse environment conditions make this species difficult to control. Moreover, the evolution of multiple herbicide resistance has reduced herbicide options for the management of common waterhemp. A synthetic auxinic herbicide (SAH), 2,4-dichloro-phenoxy acetic acid (2,4-D), has been a valuable post-emergence (POST) option to control many broadleaf weeds including common wateremp; however, the evolution of common waterhemp resistant to 2,4-D can affect the utility of 2,4-D-resistant corn and soybean. Common waterhemp resistant to 2,4-D was first documented in 2009 in Nebraska^3^, followed by Illinois^4^, and more recently in Missouri^5^. The WHR (2,4-D resistant common waterhemp) population from Nebraska is 8-10-fold resistant to 2,4-D compared to a known susceptible (WHS) population^3^. Further, a rapid metabolism of 2,4-D, possibly mediated by cytochrome P-450 monooxygenases, has been reported to confer resistance in this population^6^. Similarly, 2,4-D resistance in common waterhemp population from Missouri was also attributed to a rapid metabolism mediated by cytochrome P-450 monooxygenases^5^.

Reproductive success of common waterhemp is often attributed to its broader window of emergence^7,8^. Such emergence pattern demands a PRE (pre-emergence) followed by a POST herbicide program for effective control and to reduce crop yield loss^9,10^. Moreover, studies show increased ecological advantage to common waterhemp cohorts emerging early in the season than later^11^. Temperature is one of the critical environmental factors that can fluctuate throughout the growing season. In Kansas, the early emerging waterhemp is exposed to a lower day/night temperature ranging from 18.4–29.0/3.1–20.6 °C (d/n; average 24.7/11.6 °C), while late in the season diurnal temperatures ranges from 28.2–40.5/15.1–27.1 °C (d/n; average 34/21.2 °C)^12^. Temperature can affect the growth and development of common waterhemp^13^, which in turn can influence the efficacy of POST herbicide application^14^. Below optimal efficacy of POST-herbicide not only results in reduced weed control but can also select resistant biotypes due to increasing chances of survival and seed production.

2,4-D, is widely used for managing dicotyledonous weeds in several crops and non-crop areas. Additionally, 2,4-D choline/glyphosate/glufosinate-resistant corn (Enlist^TM^ corn) is commercially available from 2018 growing season in the United States and 2,4-D- choline/glyphosate/glufosinate-resistant soybean (Enlist^TM^ soybean) is likely to be commercially available in the near future. In sensitive dicotyledonous weeds, 2,4-D is absorbed through root, stem, and leaves and gradually translocates systemically to meristems^15^. Plant species tolerant to 2,4-D naturally degrade this herbicide into inactive metabolites, thus preventing the active ingredient to translocate further^16^. For instance, in corn, 2,4-D is metabolized via ring hydroxylation mediated by cytochrome P-450 monooxygenases^17,18^. Similar to monocotyledonous weeds, in many 2,4-D-resistant dicotyledonous weeds such as corn poppy (*Papavar rhoeas*)^19^, common waterhemp^5,6^, degradation was possibly mediated by cytochrome P-450 monooxygenases. Apart from metabolism, reduced absorption and/or translocation of 2,4-D have also been found to bestow 2,4-D resistance in several dicotyledonous weeds such as corn poppy^20^, prickly lettuce (*Lactuca serriola*)^21^ and wild radish (*Raphanus raphanistrum*)^22^.

The effect of temperature on herbicide efficacy often vary depending on weed species and herbicide site of action. For example, Ganie *et al*.^23^ found that efficacy of 2,4-D was improved to control giant ragweed (*Ambrosia trifida*) and common ragweed (*Ambrosia artemisiifolia*) at temperature 29/17 °C, d/n due to increased 2,4-D translocation compared to 20/11 °C, d/n temperature. In contrast, Ou *et al*.^24^ reported reduced control of kochia (*Kochia scoparia*) at a higher temperature (32.5/22.5 °C, d/n) compared to a lower temperature (17.5/7.5 °C, d/n) due to reduced absorption of glyphosate and reduced translocation of dicamba. Scientific literature is not existing on effect of temperature on efficacy of 2,4-D for control of 2,4-D-resistant and susceptible common waterhemp. Understanding the effect of temperature on efficacy of 2,4-D as a post-emergence option will help to better facilitate control of common waterhemp. The objectives of this research were (1) to evaluate the efficacy of 2,4-D on WHS and WHR control at a high (HT; 34/20 °C, d/n) and low (LT; 24/10 °C, d/n) temperature regimes, and (2) to investigate the uptake, translocation, and metabolism of ^14^C 2,4-D in WHS and WHR common waterhemp at aforementioned temperature regimes.

## Results

### 2,4-D dose-response experiment

**Figure 1:**
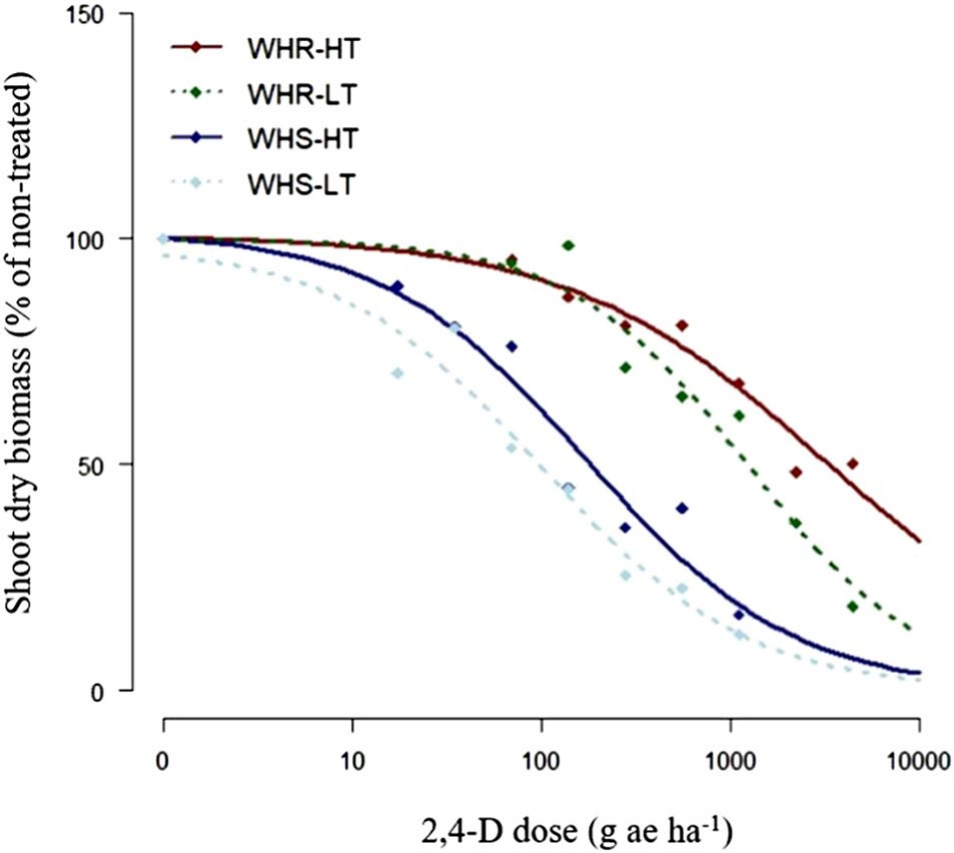
Whole-plant 2,4-D dose-response of 2,4-D susceptible (WHS) and -resistant common waterhemp (WHR) at low (LT; 24/10 °C, d/n) and high (HT; 34/20 °C, d/n) temperature regimes based on dry shoot biomass at 4 weeks after treatment (WAT).

WHS and WHR exhibited varying response to 2,4-D at HT or LT regime (Fig. 1). At 4WAT, the amount of 2,4-D required to reduce 50% (GR_50_) growth of WHS and WHR plants grown at HT regime were 178 and 3,696 g ae ha^−1^ and while at LT regime were 107 and 1,001 g ae ha^−1^, respectively (Table 1). Thus, the resistance indices of WHR relative to WHS grown at HT and LT regimes were ~20 and ~10, respectively, suggesting that WHR common waterhemp showed increased level of resistance to 2,4-D at HT compared to LT (Fig. 1, Table 1). “CompParm” function in R indicated that there is significant diference between GR_50_ of WHR at HT and LT (p < 0.05), WHR and WHS at HT (p < 0.01), WHR and WHS at LT (p < 0.001). However, there was no significant difference between GR_50_ of WHS at HT and LT. This suggstes reduction in efficacy of 2,4-D at HT to control 2,4-D-resistant common waterhemp (Fig. 1).

**Table 1:** Regression parameters estimated from the whole-plant 2,4-D dose- response study based on dry shoot biomass of 2,4-D–susceptible (WHS) and –resistant (WHR) common waterhemp grown under low (24/10°C, d/n) and high (34/20 °C, d/n) temperature regimes at 4 weeks after treatment (WAT).

Population	Temperature (°C)	Effective herbicide dose	Resistance Index (RI)	Regression parameters	
		GR_50_ (g ae ha^−1^)		b	d
WHS	24/10	107 (±26)	—	0.88 (±0.14)	99.88 (±6.00)
	34/20	178 (±43)	—	0.76 (±0.11)	101.27 (±5.80)
WHR	24/10	1001 (±237)	9.35	0.81 (±0.14)	100.39 (±5.95)
	34/20	3696 (±1138)	20.76	0.65 (±0.16)	100.57 (±5.58)

^a^Data combined from two runs.

^b^GR_50_ is the effective 2,4-D doses (g ae ha^−1^) required for 50% reduction in shoot dry biomass.

^c^RI is calculated as a ratio of GR_50_ ofthe WHR population to GR_50_ of the WHS population.

^c^Values in parenthesis are standard error of mean.

The test for ‘lack of fit’ in ‘drc’ was non-significant (p = 0.88), suggesting that the data fitted the regression model reasonably. Root means square error (RMSE) values of the 2,4-D dose-response experiments conducted at HT and LT ranged from 1.82 to 2.48 for WHS and 2.65 to 2.04 for WHS respectively, indicating a good fit.

### ^14^C 2,4-D absorption and translocation experiment

Regression analysis of ^14^C 2,4-D absorption indicated that temperature did not affect the absorption or translocation of ^14^C 2,4-D in both WHS and WHR and there was no significant difference between A_max_ (upper limit of absorption) and A_90_ (the time required to achieve 90% of maximum absorption) of WHR and WHS at HT and LT conditions. A_max_ for WHS and WHR at HT and LT regimes were 96.31 (±3.70), 92.73 (±3.61), and 93.43 (±2.54), and 95.35 (±3.16) %, respectively (Table 2). Moreover, A_90_ was also similar in WHS and WHR plants at HT or LT regimes i.e., 18 (±6.19), 13 (±7.38), 16.43 (±5.17), and 22.12 (±7.61) hours, respectively (Table 2). Similarly, there was no significant difference between T_max_ (upper limit of translocation) and T_90_ (the time required to achieve 90% of maximum translocation) between WHS and WHR at two temperature regimes, which indicated that temperature regimes did not affect ^14^C 2,4-D translocation. The predicted T_max_ for WHS and WHR at HT and LT regimes were 75.69 (±14.39), 79.18 (±14.03) and 70.83 (±14.39), and 73.78 (±18.92) %, respectively (Table 2). The time required to achieve 90% of the maximum translocation of 2,4-D in WHS and WHR plants were 111.63 (±55.07), 119.73 (±70.20) and 113.12 (±77.17), 120.59 (±94.74) hours, respectively, at HT and LT regimes (Table 2).

**Table 2:** Regression parameter estimates of ^14^C 2,4-D absorption and translocation of 2,4-D- susceptible (WHS) and -resistant (WHR) common waterhemp at low (24/10 ^o^C, d/n) and high (34/20 ^o^C, d/n) temperature regimes using rectangular hyperbola model.

Population	Temperature (°C)	Absorption		Translocation	
		A_max_	A_90_	T_max_	T_90_
WHS	24/10	92.73 (±3.61)	13 (±7.38)	79.18 (±14.03)	119.73 (±70.20)
	34/20	96.31 (±3.70)	18 (±6.19)	75.69 (±14.39)	111.63 (±55.07)
WHR	24/10	95.35 (±3.16)	22.12 (±7.61)	73.78 (±18.92)	120.59 (±94.74)
	34/20	93.43 (±2.54)	16.43 (±5.17)	70.83 (±14.39)	113.12 (±77.17)

^a^Data combined from two runs.

^b^A_max_ and T_max_ is the maximum absorption or translocation (%), A_90_ or T_90_ is the time (h) required to achieve 90% of the maximum absorption or translocation.

^c^Values in parenthesis are standard error of mean.

### ^14^C 2,4-D metabolism experiment

**Figure 2:**
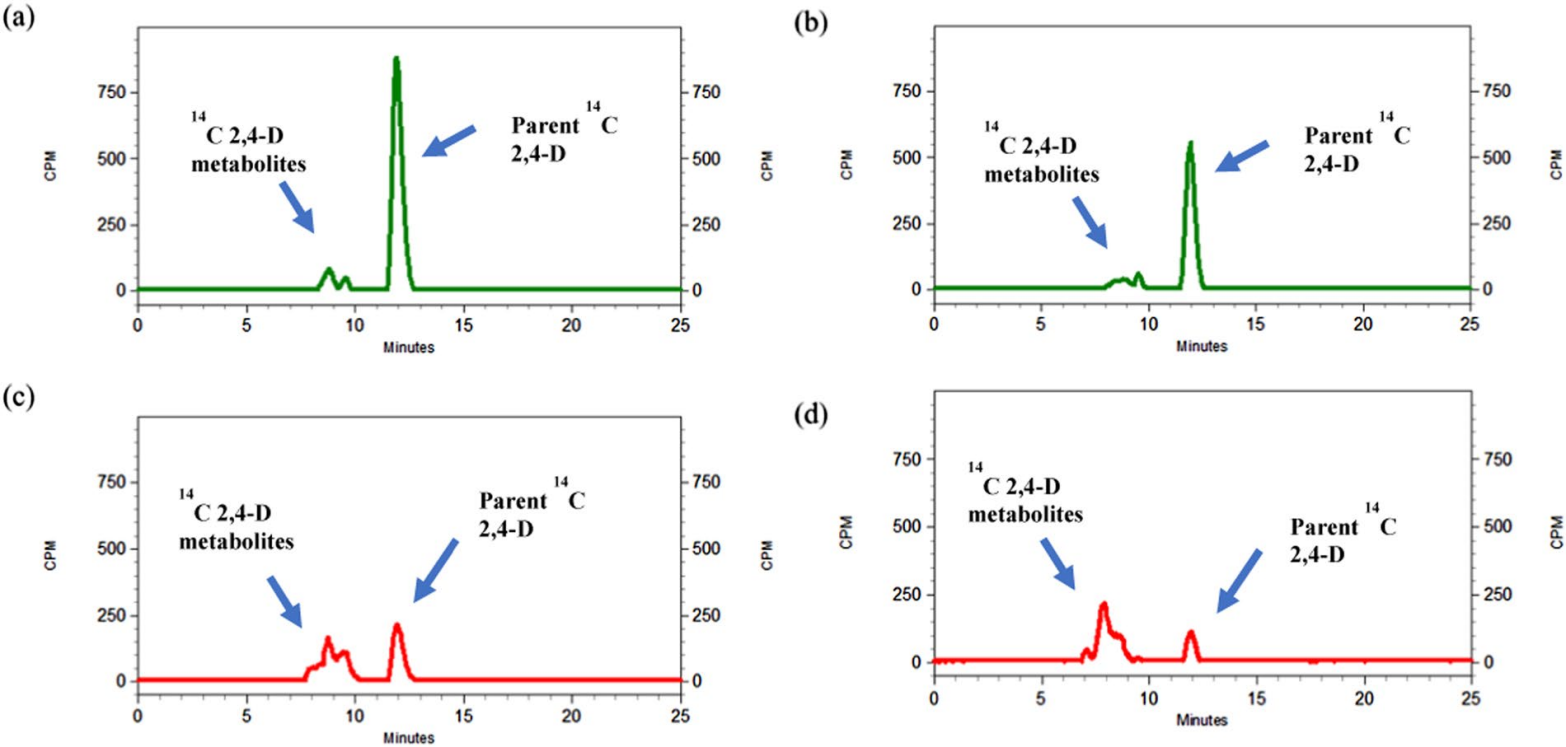
^14^C 2,4-D parent compound and its metabolites in (**a**,**b**) 2,4-D–susceptible (WHS) and (**c**,**d**) 2,4-D resistant (WHR) common waterhemp populations at 24 hours after treatment (HAT) at (**a**,**c**) low temperature regime (24/10 °C, d/n) and (**b**,**d**) high temperature regime (34/20 °C, d/n).

**Figure 3:**
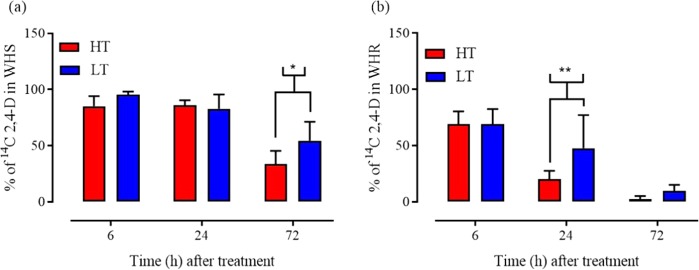
Percentage of ^14^C 2,4-D parent compound in (**a**) 2,4-D susceptible (WHS) and (**b**) resistant (WHR) common waterhemp populations at 6, 24, and 72 hours after treatment (HAT) at low (LT; 24/10 °C, d/n) and high (HT; 34/20 °C, d/n) temperature regimes. Data combined over two runs. *P-value < 0.05, **P-value < 0.001, ***P-value < 0.0001, indicates the level of significance of difference in means, and error bars represent standard error of mean).

The HPLC chromatographs indicated that the retention time of the parent ^14^C 2,4-D (used as standard) was 11.96 min (Fig. 2). Peaks of parent 2,4-D were much taller in WHR at LT compared to HT at 24 and 72 HAT. However, such difference was not observed at 6 HAT in WHR plants (Fig. 3b) At 6 HAT, the mean 2,4-D retention by WHR and WHS common waterhemp at HT and LT temperature regimes was 69.3, 69.3%, and 85.1, 95.3%, respectively (Fig. 3a,b). Twenty-four HAT, WHR plants retained 20.2 and 47.7% of parent 2,4-D at HT (Figs 2d and 3b) and LT (Figs 2c, 3b), respectively. Whereas, WHS retained 82.3 (Figs 2b and 3a) at HT and 86.1 (Figs 2a and 3a) % at LT, respectively. This validates that, metabolism of 2,4-D plays a key role in bestowing 2,4-D resistance in WHR (Fig. 2). More importantly, this indicates that at 24 HAT, WHR plants grown at LT retained approximately 27% more parent 2,4-D than at HT (Figs 2c,d and 3b). This indicates rapid metabolism of 2,4-D in WHR plants grown at HT compared to LT. Also, at 72 HAT, the WHR plants grown at HT conditions metabolized close to 100% of the parent 2,4-D while those at LT still retained 9.4% (Fig. 3b). At 72 HAT the WHS plants retained 33.7, 54.5% of parent 2,4-D at HT and LT conditions, respectively (Fig. 3a). Overall, the rate of 2,4-D metabolism increased both in WHR and WHS at HT (Fig. 3a,b).

The two-way analysis of parent 2,4-D retained in WHR followed by mean comparison using LSD (p = 0.05) suggested that there is a significant difference in % parent 2,4-D present in WHR at 24 HAT (Fig. 3b) with more 2,4-D being retained in plants grown at LT. In case of WHS plants, such difference was observed at 72 HAT (Fig. 3a) with more 2,4-D retained at LT compared to HT.

## Discussions

**Figure 4:**
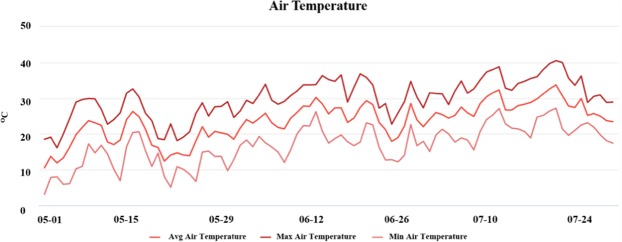
Average, maximum, and minimum air temperature in Kansas during May to July, a typical common waterhemp emergence time in the state (KSU, Mesonet 2018).

The time of emergence of common waterhemp under field conditions depends on various factors including, soil temperature, moisture, and seed dormancy. Especially, in the Midwestern United States, common waterhemp emergence occurs over a wider time frame compared to other summer annual weed species^25^. The average diurnal temperatures in May and July, the two-major seasons for waterhemp cohort emergence, are around 24/10 °C and 34/20 °C in Kansas (Fig. 4)^12^. The dose-response study results demonstrated reduced efficacy of 2,4-D at HT (34/20 °C) compared to LT (24/10 °C) for controlling both WHS and WHR common waterhemp. In contrast, Ganie *et al*.^23^ reported improved efficacy of 2,4-D or glyphosate at HT (29/17 °C) compared with LT (20/11 °C) for common and giant ragweed control regardless of susceptibility or resistance to glyphosate. Godar *et al*.^26^ reported reduced efficacy of mesotrione for Palmer amaranth (*Amaranthus palmeri*) control at high (40/30 °C) compared to low (25/15 °C) temperature due to reduced translocation coupled with rapid metabolism of mesotrione and increased 4-hydroxyphenylpyruvate dioxygenase (HPPD)-gene expression. However, as previously reported by Figueiredo *et al*.^6^ the data from this study also showed no difference in 2,4-D absorption or translocation between WHR and WHS (Table 2). The maximum limit of ^14^C 2,4-D absorption in this study was found to be 95% in WHR and WHS common waterhemp (Table 2). Previous studies have shown that 2,4-D absorption can range from 10–99% depending on several factors such as environment, weed species and other application factors^27^. Similar to our findings, Coetzer *et al*.^28^ reported no effect of temperature on glufosinate absorption in Palmer amaranth.

High temperature increased the rate of metabolism of 2,4-D both in WHR and WHS common waterhemp. Similar to these findings, Johnson and Young^29^, reported a 6–7-fold higher susceptibility of common waterhemp to mesotrione at 18 °C compared to 32 °C. Likewise, Olsen *et al*.^30^ reported decreased metabolism of MON 37500 in several grass weeds (*Aegilops cylindrica*, *Avena fatua*, *Bromus tectorum*) grown at cool air temperature. Gallaher *et al*.^31^ observed rapid metabolism of primisulfuron and nicosulfuron in broadleaf signalgrass (*Brachiaria platyphylla*) at high (30/20 °C) compared to low (20/10 °C) temperature.

The auxinic herbicide-tolerant monocotyledonous weeds are known to metabolize 2,4-D via ring hydroxylation mediated by cytochrome P-450 monooxygenases, an enzyme family predominantly involved in metabolizing xenobiotics in plants^16,32^. A possible involvement of these enzymes in 2,4-D degradation has been documented in many dicotyledonous weeds, resistant to this herbicide. For example, cytochrome P-450 mediated 2,4-D degradation has been reported in 2,4-D-resistant corn poppy^19^. Figueiredo *et al*.^6^ reported a 7-fold reduction in GR_50_ of WHR (the same common waterhemp) with pre-treatment of malathion (a cytochrome P-450-inhibitor) followed by 2,4-D compared to plants treated with 2,4-D alone, indicating a possible involvement of cytochrome P-450s in 2,4-D metabolism in common waterhemp. Thus, it is likely that a rapid metabolism of 2,4-D in WHR plants grown at HT is facilitated by increased activity of cytochrome P-450 enzymes. Previously, Viger *et al*.^33^ reported rapid metabolism of metolachlor at a high temperature (30 °C) compared to a low temperature (21 °C), which was associated with a five-fold increase in glutathione-S-transferase (GST) activity in corn. Therefore, the possible increased cytochrome P-450 enzyme activity may be an example of common waterhemp adaptation to high temperature stress. Studies have shown that plant response to stress, including abiotic stress can lead to further selection of resistant weed biotypes^34^. Hence, application of 2,4-D at the most effective temperature regime is important to control common waterhemp and reduce further selection of 2,4-D resistance.

In conclusion, the results of this research demonstrate that 2,4-D efficacy can be improved at low temperature regime (24/10 °C, d/n) to manage common waterhemp. Thus, applying 2,4-D when day temperature is lower than 30 °C is desirable for common waterhemp control; however, apart from air temperature other abiotic factors such as light intensity, relative humidity, and plant factors such as leaf orientation also play key role in affecting herbicide efficacy. Our studies were conducted in growth chambers where apart from temperature all other factors were kept constant. This is particularly important to reduce common waterhemp competition and crop yield loss and reduce selection for resistance. In general, the efficacy of auxinic herbicides for controlling dicotyledonous weeds depends on several factors including time of application^34–37^. Additionally, efficacy of 2,4-D is species dependent as improved efficacy at HT has been noticed for control of common and giant ragweed. Therefore, further studies are needed to assess the interaction of other abiotic and plant factors that can influence 2,4-D efficacy for controlling common waterhemp.

## Materials and Methods

### Plant materials and growth conditions

WHS and WHR common waterhemp from Nebraska, USA were used in this study^3,6^. Common waterhemp resistant to 2,4-D (WHR) has been confirmed in a native grass little bluestem (*Schizachyrium scoparium*) production field in southeastern Nebraska where 2,4-D was applied for over 10 years^6^. The susceptible population (WHS) was collected from a soybean field near Auburn, Nebraska^3,6^.

WHS and WHR common waterhemp seeds were germinated in plastic trays (25 × 15 × 2.5 cm) filled with potting mix (Fafard® ultra container potting mix, Sungro Horticulture, Agawam, MA). After emergence, individual seedlings at 2–3 leaf stage were transplanted into plastic pots (6 × 6 × 6 cm) and kept in the greenhouse maintained at 25/20° C day/night (d/n), 15 hours of photoperiod supplemented with 120 μmol m^−2^ s^−1^ illumination provided with sodium vapor lamps along with 60 ± 10% relative humidity. At 7 days after transplanting, half of the small and uniform seedlings (4-leaf stage) were transferred in growth chambers set at HT (34/20°C, d/n) and the rest were transferred in a separate growth chamber set at LT (24/10°C, d/n). Temperature regimes were selected based on the average diurnal temperatures during mid-May to mid-June in Kansas, USA^12^. Incandescent and fluorescent bulbs were used in growth chambers to maintain light level of 750 μmol m^−2^ s^−1^ (15/9 hrs, d/n condition) and relative humidity was maintained at 60 ± 10% throughout the study. Plants were watered daily and fertilized once a week after transplanting.

### 2,4-D dose-response experiment

Ten to 12 cm tall WHS and WHR common waterhemp plants grown at HT or LT were treated with several rates of 2,4-D (2,4-D Amine 4, Winfield Solutions, LLC, St. Paul MN, USA). Specifically, the WHS plants were treated at 0, 17.5, 35, 70, 140, 560, 1,120 g ae ha^−1^ 2,4-D whereas, the WHR plants were treated with 0, 70, 140, 280, 560, 1,120, 2,240, 4,480 g ae ha^−1^ 2,4-D, using a bench-type sprayer (Research Track Sprayer, Generation III, De Vries Manufacturing, Hollandale, MN, USA) equipped with a single flat-fan nozzle (80015LP TeeJet tip, Spraying Systems Co., Wheaton, IL, USA) delivering 187 L ha^−1^ at 220 kPa in a single pass at 3.2 km h^−1^. The treated plants were transferred back in respective growth chambers 30 min after 2,4-D application. At 4 weeks after treatment (WAT), above-ground biomass from each plant was harvested and placed in paper bags and dried in an oven at 60 °C for 72 hours (h) to measure dry shoot biomass. Percent dry shoot biomass was calculated relative to the non-treated control for each common waterhemp population as follows:

$$Shoot\,biomass\,( \% )=\frac{biomass\,of\,each\,sample\times 100}{biomass\,of\,the\,{\rm{non}} \mbox{-} {\rm{treated}}\,sample}$$

### ^14^C 2,4-D absorption and translocation experiment

WHS and WHR seedlings, raised and grown in the greenhouse (as described above) were transferred to growth chambers maintained at high (HT: 34/20°C, d/n) and low (LT: 24/10 °C, d/n) temperatures. ^14^C 2,4-D working solution was prepared by mixing ^14^C 2,4-D [3.3 kBq µl^−1^ with a specific activity of 5.5 MBq mmol^−1^ (Dow AgroSciences, Indianapolis, IN, USA)] with commercially available 2,4-D (2,4-D Amine 4, Winfield Solutions, LLC, St. Paul MN, USA) to obtain 560 g ae ha^−1^ 2,4-D in a carrier volume of 187 L. Ten to 12 cm tall (8 to10 leaf stage) plants were treated with ten 1-µl droplets of ^14^C 2,4-D working solution on the adaxial surface of the fourth youngest fully expanded leaf using Wiretrol^®^ (10 μL; Drummond Scientific Co., Broomall, PA, USA). After 30 minutes, the treated plants were returned to respective growth chambers maintained at HT or LT. The plants were harvested at 6, 24, and 72 hours after treatment (HAT), and separated into treated-leaf (TL), tissue above treated-leaf (ATL), and below treated-leaf (BTL). TL were washed with 5 ml of wash solution containing 10% (v/v) aqueous solution of ethanol and 0.5% Tween-20 in 20-ml scintillation vials for 1 minute to remove excess unabsorbed 2,4-D from the leaf surface. The leaf rinsate was mixed with 15 ml of scintillation cocktail [Ecolite-(R), MP Biomedicals, LLC. Santa Ana, CA, USA] to measure the radioactivity using liquid scintillation counter  (LSC; Beckman Coulter LS6500 Liquid Scintillation Counter, Beckman Coulter Inc., Fullerton, CT, USA). Plant sections were oven dried at 60 °C for 72 h, and then combusted for 3 min using a biological oxidizer (OX-501, RJ Harvey Instrument, Tappan, NY, USA). The ^14^C 2,4-D was recovered in a scintillation cocktail [Carbon-14 (C14) Cocktail, RJ Harvey Instrument, Tappan, NY, USA] and the radioactivity was measured using a LSC. The data was converted into percentages using the following equations^26^,

$$Percentage\,absorption\,({percentR}_{absorbed})=\frac{({R}_{applied}-{R}_{rinsate})\times 100}{{R}_{applied}}$$

$$Percentage\,translocation=(100-percent\,{R}_{TL})$$

$$Percentage\,radoiactivity\,recovered\,in\,treated\,leaf\,=\frac{{R}_{TL}\times 100}{{R}_{absorbed}}$$

In the above equations, R_absorbed_ is the radioactivity absorbed; R_applied_ is total amount of radioactivity applied on the plant; R_rinsate_ is the radioactivity recovered in leaf rinsate; and R_TL_ is the radioactivity recovered in the treated leaf (TL).

### ^14^C 2,4-D Metabolism experiment

The WHS and WHR common waterhemp plants (10–12 cm tall) grown under high and low temperature regimes (as described above) were used. The adaxial surface of the fourth youngest fully expanded leaf was treated with 10-µl droplets of ^14^C 2,4-D working solution containing ^14^C 2,4-D (5 kBq µl^−1^ with a specific activity of 5.5 MBq mmol^−1^) and commercial 2.4-D and plants were returned to growth chambers. Treated plants were harvested at 6, 24, and 72 HAT. At each harvest time, the TL was washed as described in absorption and translocation experiment to remove excess unabsorbed 2,4-D from the leaf surface. Above-ground plant tissue including the TL was wrapped in aluminum foil and flash frozen in liquid nitrogen to store at −80 °C. The frozen plant tissue was later grinded using a mortar and pestle. The ^14^C 2,4-D, and its metabolites were extracted with 15 ml of 90% aqueous acetone in a centrifuge tube and preserved at 4 °C for at least 16 hours. After 16 hours, the tubes were centrifuged at 5,000 × g for 10 minutes. The supernatant was transferred to a new centrifuge tube and concentrated at 45°C for 1.5–2 h with a rotary evaporimeter (Centrivap, Labconco, Kansas City, MO). The final volume of the supernatant was maintained around 600 µL and transferred to a 1.5 ml microcentrifuge tube and centrifuged at 10,000 × g for 10 minutes. The radioactivity of the supernatant solution was measured with the liquid scintillation counter and normalized by diluting the samples with 50% acetonitrile (1:1 v/v acetonitrile:water). The final solutions were analyzed using reversed-phase high-performance liquid chromatography (HPLC) (BeckmanCoulter system Gold 126 solvent module, Beckman Coulter Inc., Fullerton, CA, USA) to resolve the solution contents into parent ^14^C 2,4-D and its metabolites.

### Experimental design and statistical analysis

The experiments were arranged in a split-plot design with four replications and repeated in time. Growth chambers were switched between two experimental runs to avoid effect of growth chamber on plant response. The dose-response experiments were arranged in a two-way factorial combination of temperature regimes (HT and LT) as main factor and herbicide doses for each common waterhemp population as sub-plot factor.

Relative shoot biomass data obtained from the whole plant dose-response study were analyzed using the ‘drc’ package (drc 1.2, Christian Ritz and Jens Strebig, R2.5, Kurt Hornik, online) in R (R statistical software, R Foundation for Statistical Computing, Vienna, Austria; http://www.R-project.org) as per Knezevic *et al*.^38^ A dose-response regression model was constructed using the three-parameter log-logistic equation.

$$Y=\{\frac{d}{1}+\exp [b(logX-loge)]\}$$

In equation above, Y is response variable (% reduction in biomass compared to control), b denotes relative slope around e, e is GR_50_ (effective dose to reduce biomass of the population by 50%) and d is the upper limit of the model. The ratio of GR_50_ values of WHS and WHR common waterhemp in HT and LT conditions were calculated to determine the level of resistance or the resistance index. Estimated GR_50_ values were then compared with each other using the “compParm” function in ‘drc’ package in R.

Fitness of the log-logistic regression model used above was assessed through the “Lack-of-fit” test in ‘drc’ using “modelFit” function. Further, root mean square error (RMSE) was calculated to test the goodness of fit of the data. The formula used for RMSE^25^ was:

$$RMSE=[\frac{1}{n}\mathop{\sum }\limits_{i=1}^{n}{({P}_{i}-{O}_{i})}^{2}]$$

where, n is the number of observations and O_i_ and P_i_ are the observed and predicted value of the observations respectively.

Absorption, translocation, and metabolism experiments, treatments were arranged in a two-way factorial combination with temperature regime (HT and LT) as the main-factor and harvesting time (6, 24, and 72 HAT) as sub-factor for each common waterhemp population. The percentage of herbicide absorbed and translocated were used to fit asymptotic regression, rectangular hyperbola (RHB), and linear model according to Kniss *et al*.^39^ using ‘drc’ and ‘*qPCR*’ packages in R. After fitting the data to these three models, the bias-corrected Akaike information criteria (AICc) of each model was obtained and compared. For analyzing both 2,4-D absorption and translocation, the RHB model was selected due to the lowest AICc values. The RHB model used is:

$$Absorption=\frac{({A}_{max}\times t)}{[(10/90)\times {A}_{90}+t]}$$

$$Translocation=\frac{({T}_{max}\times t)}{[(10/90)\times {T}_{90}+t]}$$

In the above equations, absorption is percent herbicide absorbed expressed in terms of percentage herbicide applied to the plant, A_max_ is the maximum herbicide absorption in time t, and A_90_ is the time required for 90% of the absorption to occur. Similarly, translocation is the percent herbicide translocated expressed in terms of percentage herbicide absorbed in the plant, T_max_ is the maximum herbicide translocation in time t, and T_90_ is the time required for 90% of the translocation to occur. A_max_, A_90_, T_max_, and T_90_ parameters of WHR and WHS at each temperature regime were compared using the “compParm” function in the ‘drc’ package.

In metabolism experiments, chromatographs obtained from HPLC profiling were used for visual assessment of ^14^C 2,4-D degradation. Percent parent ^14^C 2,4-D present in each sample was determined and analyzed using GraphPad Prism 7.04® (GraphPad Software, San Diego, CA) at p = 0.05 and comparisons were made between HT and LT conditions in each biotype.
